# Long-term sediment decline causes ongoing shrinkage of the Mekong megadelta, Vietnam

**DOI:** 10.1038/s41598-020-64630-z

**Published:** 2020-05-15

**Authors:** Toru Tamura, Van Lap Nguyen, Thi Kim Oanh Ta, Mark D. Bateman, Marcello Gugliotta, Edward J. Anthony, Rei Nakashima, Yoshiki Saito

**Affiliations:** 1 Geological Survey of Japan, AIST, Tsukuba Ibaraki, 305-8567 Japan; 2 Graduate School of Frontier Sciences, The University of Tokyo, Kashiwa Chiba, 277-8561 Japan; 3 HCMC Institute of Resources Geography, VAST Ho Chi Minh City, Vietnam; 4 Department of Geography, University of Sheffield, Sheffield, S10 2TN UK; 5 Estuary Research Center, Shimane University, Matsue, Shimane 690-8504 Japan; 6 Aix Marseille Univ, CNRS, IRD, INRA, Coll France, CEREGE, Aix-en-Provence, France; 7 USR LEEISA, CNRS, Cayenne, French Guiana

**Keywords:** Environmental sciences, Environmental social sciences

## Abstract

Since the 1990s the Mekong River delta has suffered a large decline in sediment supply causing coastal erosion, following catchment disturbance through hydropower dam construction and sand extraction. However, our new geological reconstruction of 2500-years of delta shoreline changes show that serious coastal erosion actually started much earlier. Data shows the sandy coast bounding river mouths accreted consistently at a rate of +2 to +4 km^2^/year. In contrast, we identified a variable accretion rate of the muddy deltaic protrusion at Camau; it was < +1 km^2^/year before 1400 years ago but increased drastically around 600 years ago, forming the entire Camau Peninsula. This high level of mud supply had sharply declined by the early 20th century after a vast canal network was built on the delta. Since then the Peninsula has been eroding, promoted by the conjunction of mud sequestration in the delta plain driven by expansion of rice cultivation, and hysteresis of long-term muddy sedimentation that left the protrusion exposed to wave erosion. Natural mitigation would require substantial increases in sediment supply well above the pre-1990s levels.

## Introduction

Coastal lowlands of river deltas, along with their usually humid climate, are characterized by high food productivity that supports large populations. They are also highly vulnerable to climate and sea-level changes as well as the impacts of human activities^1–3^. The Mekong delta, southern Vietnam, is the third largest delta in the world and has been characterized by long-term large sediment supply in its extensive catchment under the influence of the rainy Asian monsoon climate. However, recent anthropogenic catchment disturbances such as upstream hydropower dam construction^4,5^ and fluvial sand mining^6–9^, possibly along with climate change-induced discharge reduction^10^, are considered to have caused severe decline in the sediment discharge to the coast, compounding concerns raised by exacerbated subsidence due to accelerated groundwater extraction^11^ and thus leading to alarms being raised regarding the increasing vulnerability of this populous megadelta. This declining sediment supply has led to enhanced coastal erosion with high-resolution satellite images from 1993 to 2013 indicating a substantial increase in the last 10 years^12^. Longer-term analysis covering 1973–2015 also confirmed accelerated coastal erosion in the last few decades, particularly after 2005, in response to the increased capacity of hydropower dams in the Mekong’s catchment^13^ and intensive sand mining in the deltaic channels^6–9^.

While the fine-tuned characterization of the delta’s recent shoreline changes has highlighted the impact of industrial activities within the Mekong catchment after the 1990s, a further question persists. The exacerbation of coastal erosion after the 1990s mainly concerned the sandy river mouth area of the delta. The muddy coast along the Camau Peninsula, which forms the bulk of the large Mekong delta protrusion into the East Sea (or South China Sea), has been eroding since the 1970s^13,14^, with historical maps indicating that erosion may have started prior to 1940^15^. The ongoing shrinkage of the Mekong delta therefore cannot simply be attributed to the direct impact of recent industrial activities within the Mekong catchment. We contend that a long-term perspective using the sedimentary record is indispensable to understand the underlying causes of the Mekong delta’s shrinkage.

Long-term shoreline changes of the Mekong delta have only been constrained in the river mouth area^16^ and relevant data have been missing in the southwestern delta protuberance, which has led previous studies to make assumptions on delta growth/erosion in this area^17,18^. We hereby reconstruct the entirety of the East Sea shoreline of the Mekong delta by compiling newly obtained sediment and landform records with existing data. The reconstruction indicates temporal and spatial variations in shoreline change and highlights a close link between the onset of secular erosion and irrigation canal network development on the delta, compounded by hysteresis of the longer-term rapid muddy accretion.

## Study Area

**Figure 1:**
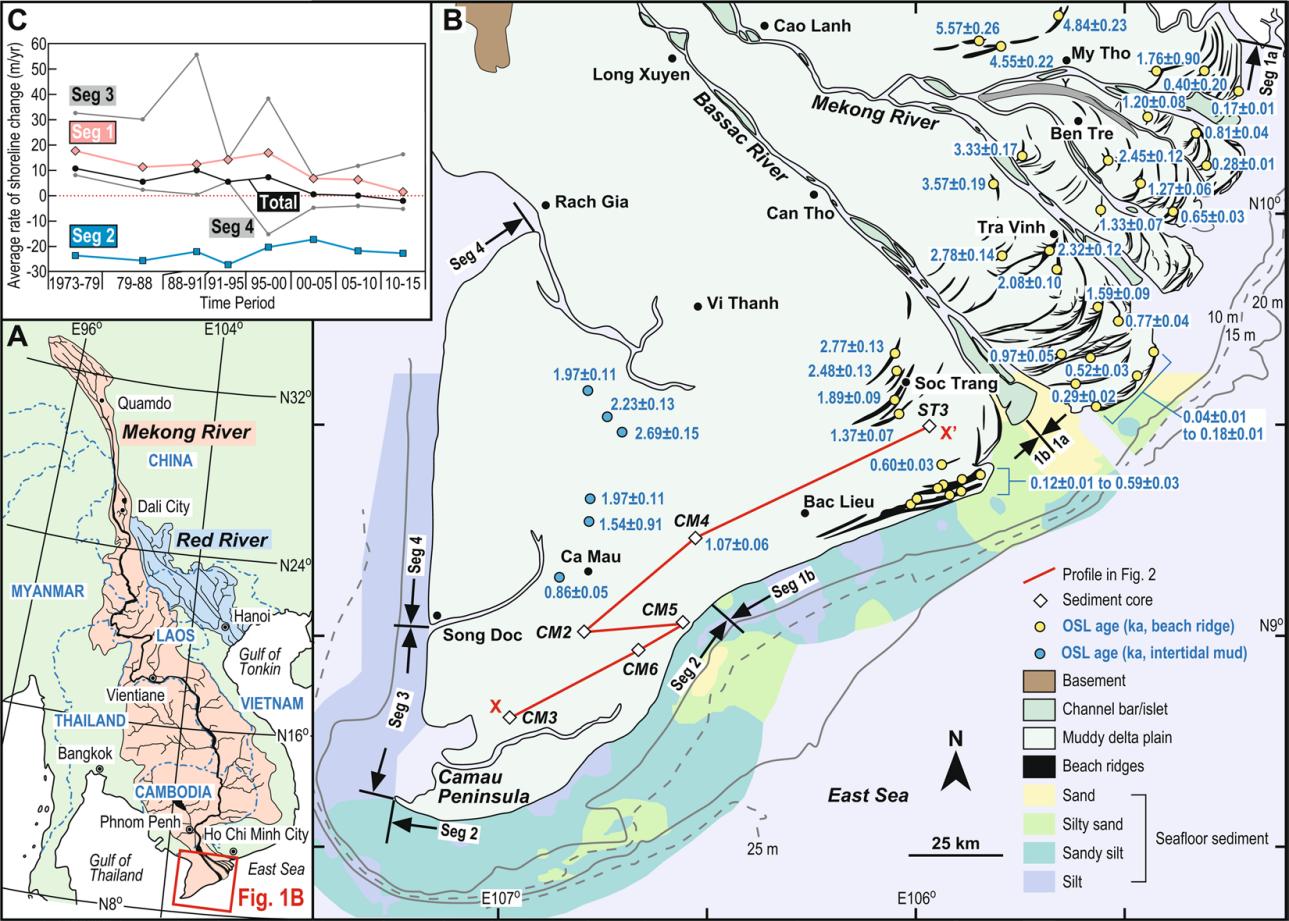
(**A**) Location of the Mekong River delta. Catchments of the Mekong and Red rivers are shaded. The map was derived from ref. ^19^ and generated with the software Adobe Illustrator 2020. (**B**) A map compiling geomorphology of the Mekong River delta^45^ and bathymetry relative to mean sea level and substrate of the coastal area^21,30^. A transect (X-X’) is defined by linking six newly obtained drill cores for the cross section in Fig. 2. OSL ages NE and SW of the Bassac River are from existing^16^ and newly reported data in this paper, respectively. Shoreline segments are defined following previous spatial analysis^13^ while Seg 1 is further divided here into Segs 1a and 1b by the mouth of the Bassac River. The map was simplified from ref. ^45^ and generated with the software Adobe Illustrator 2020. (**C**) Temporal variations of average rates of shoreline change from 1973 to 2015 for shoreline segments^13^.

The Mekong delta has prograded over the last 8000 years in response to the deceleration of the post-glacial sea-level rise^19,20^ and is a representative of a mixed-energy delta^21,22^. At present, tide-modulated wave processes dominate the open coast^22,23^, whereas distributary channels are characterized by an upstream to downstream transition from fluvial to tide-dominated estuarine processes^24–26^. Contrasts between the winter and summer monsoons define an annual sedimentary cycle on the coast^23,27,28^. The southwesterly summer monsoon is characterised by a weaker wind and wave regime but brings moisture that causes river flooding and fluvial sediment supply, whereas the northeasterly winter monsoon is drier with limited fluvial sediment supply but has a stronger wind and wave regime. Consequently, along the NE-SW trending East Sea coastline, a significant fraction of sediments deposited and stored near the river mouths in summer are reworked in winter and transported southwestwards by the wave-generated longshore current. This is aided by tidal currents^12,22,29^ and there is also a clear sediment segregation. Sandier sediments are dominant from the river mouths to about 30 km downdrift (westward) of the Bassac River mouth, the most westward of the mouths, while further downdrift the coast is composed mainly of mud^30^ (Fig. 1B). This segregation is also recorded in the relict deltaic landforms as sandy beach ridges only occur in the river mouth area^16^, whereas the Camau Peninsula consists almost entirely of a muddy delta plain.

Four shoreline segments (Segs 1–4) in the delta were defined from spatial analysis spanning from 1973 to 2015^13^ (Figs. 1B, C). Seg 1, occurring at the river mouth area from the NE end of the shoreline to 30 km SW of Bac Lieu, showed progradation during most of the interval with decreasing accretion and a switch to erosion between 2010 and 2015. Seg 2, stretching from the SW end of Seg 1 to the tip of the Camau Peninsula at Camau Point, has retreated consistently at a rate > 20 m/year. Seg 3, a complex coastline from Camau Point to an inlet at Song Doc, showed large variations associated with a gradual decrease in the net accretion rate. Seg 4, between Song Doc and Rach Gia, showed the least accretion among the four segments until early 1990 when erosion started to occur. Here, the Seg 1 shoreline is further subdivided into Segs 1a and 1b at the mouth of Bassac River (Fig. 1B).

## Results

**Figure 2:**
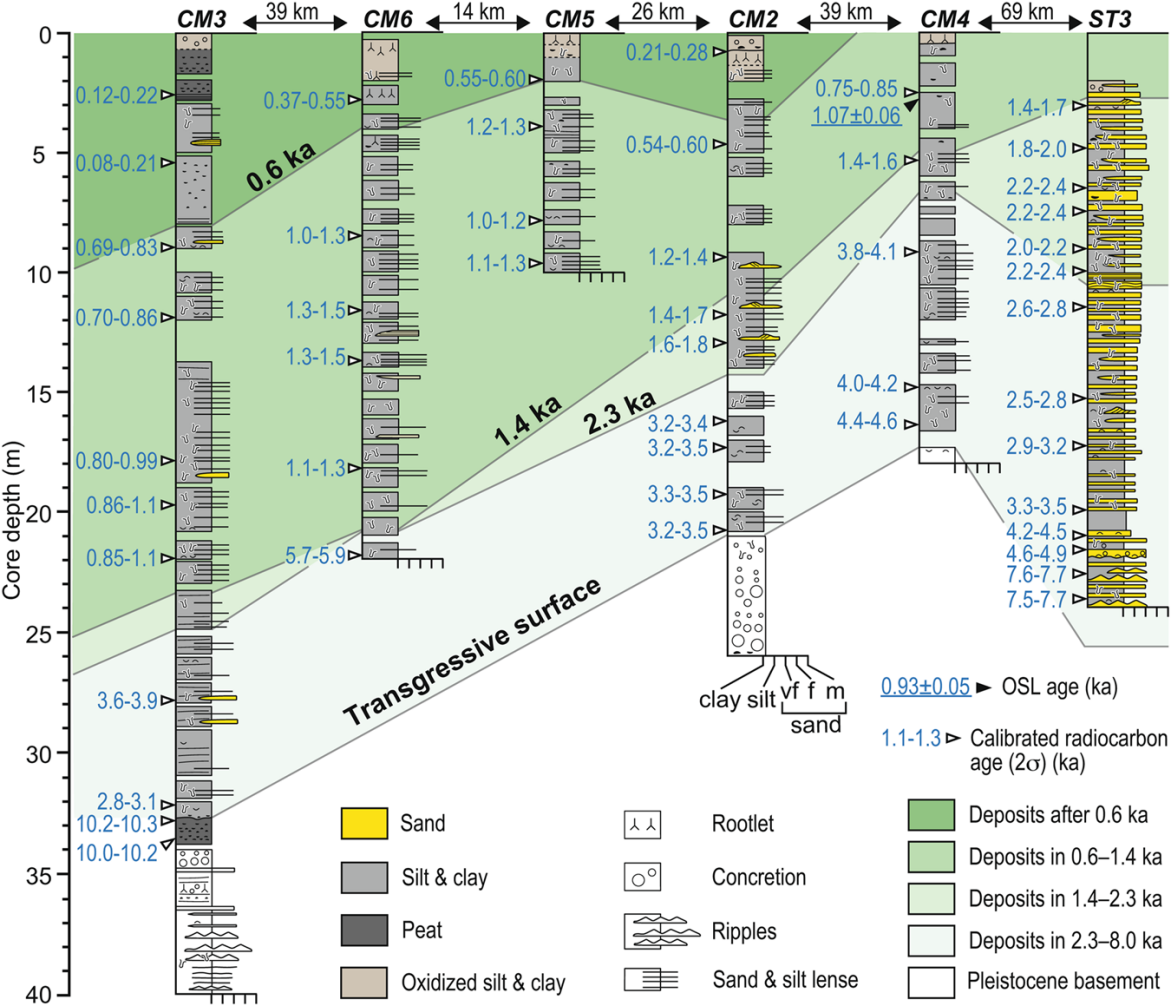
A profile defined by columnar sections of drill cores along the transect X-X’ in Fig. 1. Isochrones of 2.3, 1.4, and 0.6 ka are defined according to linear interpolation of radiocarbon ages obtained from Holocene deposits overlying the Pleistocene basement. Core depth is relative to the ground elevation at the individual site.

The sediment cores, full descriptions of which are given in the Supplementary Information, define a cross section through the delta (X-X’ in Fig. 1B). Three cores revealed the marine Pleistocene basement sediments beneath the modern delta at depths 17–34 m (Fig. 2). One core (CM3) recorded mangrove sedimentation above this basement during the post-glacial sea-level rise around 10 ka (ten thousand years ago). All other core sediments were deltaic sand and mud with marine mollusc shells and varying degrees of bioturbation. Of note, lateral fining of the sediment facies due to longshore transport was found in core ST3 which exhibited rhythmic alternations of fine to very fine sands and mud, as well as in downdrift cores CM2–6 which showed massive mud intercalated with thin lenses of coarse silt and very fine sand. This corroborates the downdrift segregation identified along the coast and delta front (Fig. 1B).

Radiocarbon and OSL dating provide the chronological framework for events (Figs. 1B and 2). Core ST3 is characterized by continuous accumulation from 4.4 to 1.4 ka. Cores CM2–6 are younger both seawards and downdrift, consistent with the southwestward progradation of the delta. Cores CM2 and CM4 represent sediment accumulation from 4.5 to 3.0 ka, an early stage of the Mekong delta progradation, while all cores record little accumulation for the interval 2.3–1.4 ka. Rapid progradation then occurred after 1.4 ka in cores CM2–6. The upper part of cores CM2, 3, 5 and 6 are dated younger than 0.6 ka, revealing that the shoreline was situated just landward of sites CM2 and CM5 at 0.6 ka. The beach ridges near Soc Trang are dated 1.4–2.8 ka, representing sandy shoreline progradation while offshore sedimentation took place at site ST3 (Fig. 1B). Seaward of Soc Trang beyond a 20-km-wide muddy plain, another beach ridge cluster is dated <0.6 ka. The two beach-ridge sets indicate two phases of slow coastal progradation at 2.8–1.4 ka and after 0.6 ka, between which occurred rapid progradation at 1.4–0.6 ka. OSL ages of intertidal sediments in the muddy plain between Camau city and Vi Thanh range from 0.9 to 2.7 ka and become younger downdrift and seawards, consistent with the shoreline progradation.

**Figure 3:**
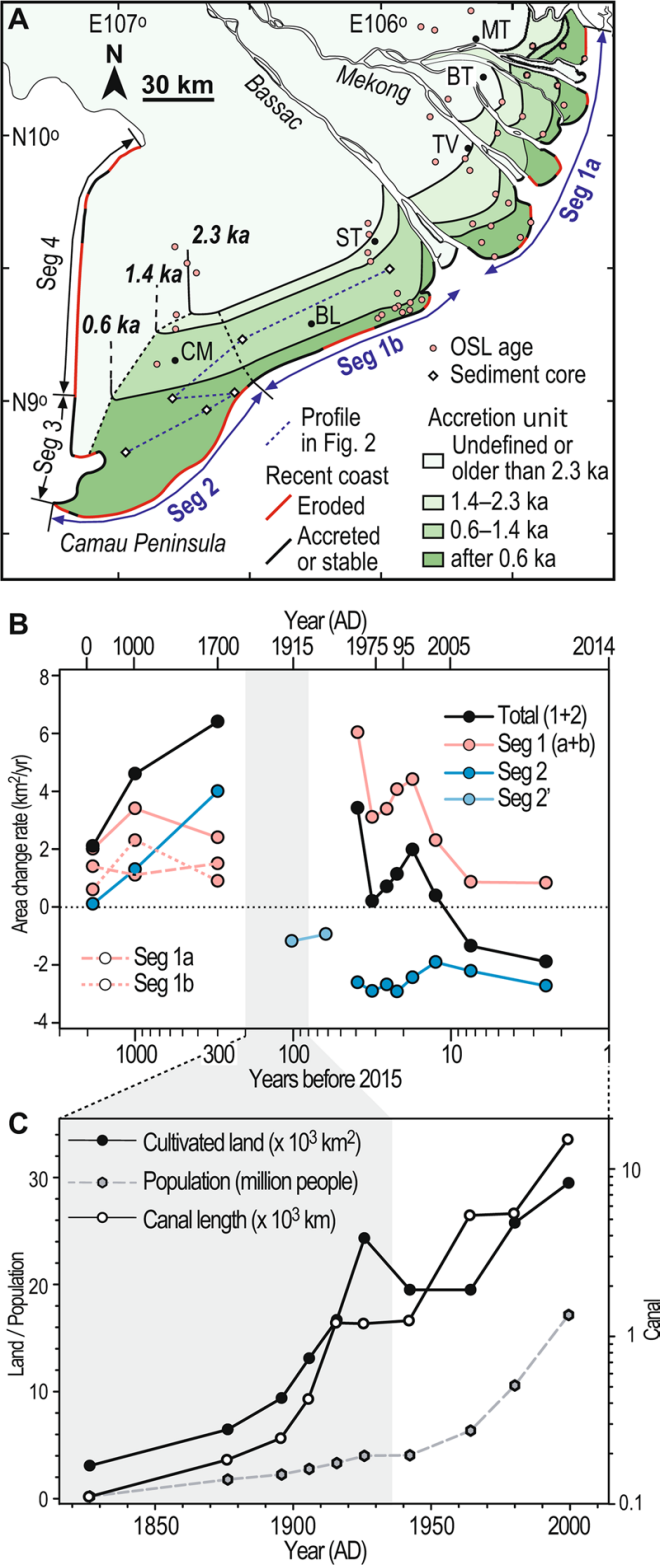
(**A**) Reconstructed past shorelines of the East Sea coast of the Mekong delta at 2.3, 1.4, and 0.6 ka based on the integration of radiocarbon ages of sediment cores and OSL ages of beach ridges and intertidal mud. ST: Soc Trang, BL: Bac Lieu, CM: Camau, TV: Tra Vinh, BT: Ben Tre, MT: My Tho. Trends of recent shoreline changes are indicated. The map was simplified from Fig. 1B and generated with the software Adobe Illustrator 2020. (**B**) Estimate of long-term area change rate for shoreline segments defined in Fig. 3A after 2300 years ago along with short-term rates over recent decades^13,15^. Seg 2′ represents the eastern half 60-km-long interval of Seg 2. Rates shown at AD 1913, 1953, 150, 1000, and 1700 represent the average of periods AD 1885–1940, AD 1940–1965, 1.4–2.3 ka, 0.6–1.4 ka, and after 0.6 ka, respectively. Grey shading indicates the period when the canal network was extensively built for irrigation of the Mekong delta under the Vietnamese kingdom and French colonial periods^41^. (**C**) Changes of the cultivated area, population, and total canal length of the Mekong delta over the last 200 years after the existing data compilation^43^.

Integration of these new and existing data^16^ enables, for the first time, reconstruction of isochrones and shoreline positions at 0.6, 1.4, and 2.3 ka along the full stretch of the East Sea coast of the Mekong delta (Fig. 3A). From these we calculated accretion rates for segments 1 and 2 (Fig. 3B). The overall accretion rate of Seg 1 has been consistent over the last 2.3 ka, ranging from +2 to +4 km^2^/year (except for 1973–77 and after the 1990s). In contrast, Seg 2 shows a drastic increase in the accretion after 1.4 ka, in phase with that of Seg 1b. However, in contrast to Seg 2, after 0.6 ka, accretion of Seg 1b appears to have slowed down whilst that in Seg 2 underwent a dramatic acceleration up to +4 km^2^/year to form the entire Camau Peninsula. A cartographic analysis^15^ has shown that over the period 1885–1940 the eastern 60-km-long fraction of Seg 2 switched to erosion at an average coastal loss rate of −1.5 km^2^/year. This has evolved into the consistent erosion of the whole Seg 2 at −2– −3 km^2^/year^13^. The total budget of Segs 1 and 2 delta area was slightly positive from the 1970s to 1990s before declined shoreline progradation in Seg 1 caused net shrinkage of the delta. Two important outcomes of the above are that consistent erosion in Seg 2 has been responsible for much of the eroded shoreline in terms of area, and that a drop of c. 6–7 km^2^/year in the sediment budget has occurred sometime before the 1970s relative to the average budget for the last 600 years.

## Discussion

The SW coast of the Mekong delta in the East Sea experienced drastic changes in the rate and pattern of progradation over the last 2.5 ka, finally reaching a trend of consistent erosion in the 20th century. Increase in sediment supply after 1.4 ka to Segs 1b and 2 could reflect both an increase in the total muddy sediment load and intensification of longshore transport. This was simultaneous with the enhanced sediment supply to the Chinese rivers by land-use changes in China^31^. These land-use changes are deemed to have accelerated the progradation of the Red River after 1–2 ka^32^, and this probably also happened in the neighbouring Mekong’s catchment (Fig. 1A). The apparent acceleration of sediment supply after 0.6 ka may also have been favoured by the Chinese population immigration to Yunnan in relation to the Min Dynasty established in the late 14th century. Climate changes, as indicated recently^10^, may account for the changes in the sediment discharge. Proxy records in South China^33^ and Tibet^34^ indicate increased regional humidity after 1.5–2 ka. The change in the Mekong delta accretion pattern at 0.6 ka, from uniform progradation of Seg 1b and Seg 2 to selective progradation of Seg 2, indicates sediment bypassing in Seg 1b. This bypassing could be accounted for by intensification of the winter monsoon that drives the longshore transport. Such an intensification has been reported at the onset of the Little Ice Age in East and Southeast Asia^35^.

Recent dam constructions, fluvial sand mining, and a local decline in mangroves^36^ cannot account for the current erosion of the eastern coast of the Camau Peninsula and for the correlative erosional features identified in the nearby subaqueous delta^30^. Our data reveal that the significant decrease in the sediment supply and ensuing erosion likely started between 1885 and 1940. We note that the average recession rate in this period appears similar (c. 20 m/year) to that after the 1970s^15^. In contrast to modern recession of the sandy coast, we propose that the causal factor for erosion at this time was sediment sequestration within the deltaic plain due to large-scale anthropogenic modification there. Flood discharge and floodplain inundation have been significantly modified by the construction of a dense network of canals and dikes^37–40^. This was promoted during the French colonial period from 1858 for navigation and agricultural irrigation^41,42^. The canal network enabled a drastic increase in the area of rice cultivation on the delta attaining up to 24,000 km^2^ by 1930^43^ (Fig. 3C). Flood waters were encouraged through the canal network to overflow into rice fields to deposit fertile sediment^37,44^. This sedimentation, augmented by sediment deposited in the canals, intercepted and stored muddy sediment in the delta plain that would otherwise have been transported to the coast^40,41^. A mean delta-plain sedimentation rate of 6.86 kg/m^2^ (6 mm/year) has been calculated from field monitoring of a delta area of about 115 km^2^ comprising a dense network of canals^39^. The normally stagnant water within these fields promotes complete sedimentation of fertile nutrients such that less sediment reaches downstream localities^40^, and, by inference, the coast. If this rate applied to the entire area of rice cultivation by the year 1930, this should have led to a huge decline in coastal mud supply. Whilst the emergence of the canal network impacted on the coastal mud supply, the bedload transported sand in delta distributaries remained unaffected. As a result, the sandy coast corresponding to Seg 1 continued progradation until the 1990s whilst the muddy Seg 2 coast eroded rapidly. It is noteworthy, however, that even Seg 1 has shown signs of a slow-down in progradation since 2003^12^. Finally, the hysteresis of longer-term sedimentation also appears to have created conditions that have ultimately favoured coastal erosion. The very rapid progradation of the Camau Peninsula after 0.6 ka enhanced its exposure to waves and longshore currents driven by the winter monsoon winds, and this has led to its increased vulnerability to erosion. This erosion^29^ can be further exacerbated by aggravated land subsidence notably related to water extraction that drastically increased after the 1990s^11^ and by ongoing change in sea levels. The extent to which sand mining and consequent deepening of the Mekong delta distributary channels^6^ affect mud redistribution is another issue for further work. Channel deepening may have a possible feedback effect on saline intrusion and re-introduction of mud up-estuary in the dry season that could potentially diminish the amount of mud transported along the eastern coast towards the Camau Peninsula^12^. The implications of our study are that even if sediment supply was restored to pre-1990s levels this would be insufficient to prevent further erosion of the Mekong’s muddy shorelines.

## Methods

To constrain the long-term deltaic sedimentation record and shoreline changes, we obtained six sediment cores, 66 radiocarbon ages, and 13 and 7 optically-stimulated luminescence (OSL) ages for beach-ridge and muddy-plain sediments, respectively. Sediment cores obtained at sites ST3 and CM2–6 (Fig. 1B, Table S1) were halved vertically and described for identification of sediment facies and collection of mollusc shells and plant fragments for radiocarbon dating. Sediment samples for OSL dating were collected by a hand auger at depths of 0.87–2.05 m, except for one taken at 2.6–2.65 m depth in core CM-4. Levels of auger samples correspond to the upper foreshore to basal foredune levels at beach ridge sites and to the intertidal level of the muddy plain, and thus are considered to represent the shoreline deposition. The cumulative subsidence of the sample sites is modeled as <0.3 m^11^ and thus insignificant. Analytical details of radiocarbon and OSL dating are provided in the supporting information. OSL ages and calibrated radiocarbon ages are expressed relative to AD 2015.

## Supplementary information

Supplementary information.
