# Supplementary material for: Long-term sediment decline causes ongoing shrinkage of the Mekong megadelta, Vietnam
Source: Sci Rep. 2020 May 15;10:8085. doi: 10.1038/s41598-020-64630-z (PMC7228934; doi:10.1038/s41598-020-64630-z)
Supplement: Supplementary file 1 — Supplementary information. [file 41598_2020_64630_MOESM1_ESM.pdf]

Supplemental Information for

Long-term sediment decline causes ongoing shrinkage of the Mekong megadelta, Vietnam

Toru Tamura[1,2], Van Lap Nguyen[3], Thi Kim Oanh Ta[3], Mark D. Bateman[4], Marcello Gugliotta[5], Edward J. Anthony[6,7], Yoshiki Saito[1,5]

1 Geological Survey of Japan, AIST, Tsukuba, Ibaraki 305-8567, Japan

2 Graduate School of Frontier Sciences, The University of Tokyo, Kashiwa, Chiba 277-8561, Japan

3 HCMC Institute of Resources Geography, VAST, Ho Chi Minh City, Vietnam

4 Department of Geography, University of Sheffield, Sheffield S10 2TN, UK

5 Estuary Research Center, Shimane University, Matsue, Shimane 690-8504, Japan

6 Aix Marseille Univ, CNRS, IRD, INRA, Coll France, CEREGE, Aix-en-Provence, France

7 USR LEEISA, CNRS, Cayenne, French Guiana

Contents of this file

Text S1

Table S1: details of drill core sites

Table S2: radiocarbon dating results

Table S3: OSL dating results

Figure S1: results of preheat dose recovery test

Figure S2: examples of equivalent dose distribution

Introduction

This supporting information provides descriptions and interpretations of sediment cores, details of the core sites, and radiocarbon and optically-stimulated luminescence (OSL) dating.

Text S1

Description and interpretation of sediment cores

We identified a succession of Holocene deltaic sandy and muddy sediments overlying the Pleistocene basement in six sediment cores obtained at sites ST3 and CM2–6 (Figs. 1B and 2, Supplementary Information Table S1). Descriptions and interpretations of these cores, aided by radiocarbon dating (Supplementary Information Table S2) for chronology, are provided below.

Core ST3

Core ST3 is 24 m long and in contrast to the other five cores consists of well-defined alternations of sand and mud. The basal 2-m interval is distinct from the rest of the succession in terms of consolidation and colour. It exhibits well-defined rhythmic alternations of very fine sand and mud with minor occurrences of burrows and shell fragments. Sand beds are 1–20 mm thick and exhibit parallel and ripple lamination. Mud layers are 1–20 mm thick and massive. Sand-mud ratios range from 1:1 to 2:3. Sporadic burrows are 5–10 mm in diameter and characterized by a thin outline of mud and filling of sand. The colour of this interval is olive brown in sand and greenish to dark greenish grey in mud whereas the rest of the succession is dark to very dark grey except for the upper most 1.5-m interval which is reddish grey. Five radiocarbon ages determined for this interval range from 7.5 to 7.9 ka (except for an older age from mollusk shell considered as reworked material; Supplementary Information Table S2). As the rhythmic alternation suggests tidally-influenced deposition, the basal interval is interpreted as estuarine or deltaic deposits formed in relation to the latest transgression to early regression of the Mekong delta shoreline during the early Holocene. Around a depth of 22 m, the basal interval is overlain by a 20-cm-thick layer of poorly-sorted, muddy very fine sand containing abundant shell fragments and grains of nodules. This poorly-sorted layer is then overlain by a 16-m-thick succession of coarse-silt/sand and mud alternations containing shell fragments and moderate bioturbation. This succession exhibits an upward-coarsening trend in two manners. The grain size of coarser layers intercalated with mud coarsens upwards from coarse silt to lower fine sand. Besides, the sand-mud ratio increases upwards from 2:8 to 9:1. Layers of sand and coarse silt also thicken upwards from 2–5 mm to 1–30 cm. Due to bioturbation, sedimentary structures in sand are generally not well defined, but where identifiable, they are characterized by parallel and ripple lamination. Various burrows are identified and most commonly sand-filled with a thin mud outline or just sand-filled with no outline. The burrow sizes range

from 5 to 30 mm in diameter. A similar 3.5 m-thick alternation of sand and mud overlies the upward-coarsening succession at 5.5 m depth but with a slight upward-fining trend from lower fine sand to lower very fine sand. Thirteen radiocarbon ages were determined for the upward-coarsening to fining succession and they are basically consistent with the stratigraphic order. These ages show regressive deposition in relation to the stillstand and slight fall of relative sea level after 5 ka. The consistent upward-coarsening trend up to the depth of 5.5 m represents the progradation of the prodelta and deltafront slope, and the coarsest interval is inferred as the interface between the deltafront platform and slope, where wave energy is attenuated through breaking. The boundary between the poorly-sorted layer and underlying basal interval around 22 m deep was not recovered. However, this boundary is considered as the ravinement surface as it represents a gap in radiocarbon age around the time interval spanning the transgression to regression and the poor-sorted facies exhibits features of lag deposits. The transgressive surface, defined at the base of the first occurrence of the Holocene marine deposits, should occur below the base of core ST3.

Cores CM2–6

Cores CM2–6 are 10–40 m long (Supplementary Information Table S1) and mostly consist of unconsolidated mud. Cores CM2, 3, and 4 reached the Pleistocene basement which is clearly distinguished from the overlying Holocene succession. While the Holocene succession is unconsolidated and mostly grey to dark grey, the Pleistocene basement is well-consolidated mud and sand coloured greenish grey with reddish yellow to reddish brown patches indicating oxidization. The sediment facies of the Pleistocene succession ranges from massive mud to rhythmic alternations of rippled fine sand and mud suggestive of marine deposition. All cores missed the interval representing the contact between the Pleistocene and Holocene successions. The upper part of the Pleistocene succession at a depth of 17.42 m in core CM4 contains a mollusk shell in burrows dated 4.7–4.9 ka (Supplementary Information Table S2), but the shell is considered to derive from the overlying Holocene deposits due to bioturbation. In core CM3, a 1-m-thick layer of peat overlies the Pleistocene basement. This layer is parallel-laminated, organic clay to silt with abundant plant fragments up to 5 cm long. The radiocarbon ages of these plant fragments are consistent, ranging from 9.8–10.0 to 10.2–10.3 ka. Further micropaleontological analysis has not been practiced for

constraining the origin of the peat layer. However, according to the relative sea-level curve in this region (Hanebuth et al., 2011), it is likely to be mangrove deposits. The upper boundary of the peat layer is sharp.

In all cores, the rest of the Holocene succession, except for the uppermost 0.5–3 m thick interval, is monotonous, massive mud intercalated with thin lenses of coarse silt and very fine sand. Lenses of coarse silt and very fine sand are commonly a few millimetre thick. Only a few layers are up to 2–3 cm thick and such layers typically show parallel and/or ripple lamination. In contrast to the systematic trend observed in core ST3, it is not possible to discern vertical trends in grain size and thickness in cores CM2–6. Burrows are ubiquitously identified as patches commonly 2–10 mm in diameter and filled with coarse silt and very fine sand. The succession contains plant fragments and shallow-marine mollusk shells, such as *Veremolpa micra*, *Barbatia virescens*, and *Moerella jedoensis*. The interval 1–3 m deep in core CM3 is a peat layer composed of organic clay to silt with abundant plant fragments. Again, the origin of this peat layer has not been constrained by micropaleontology but is likely from mangroves because of its proximity to the mean sea level. The uppermost interval of all cores is oxidized and contains rootlets. Shell and plant fragment samples from the Holocene succession, except for the basal peat in core CM3, are dated younger than middle Holocene and define a younging trend towards the southwest. These features indicate the regressive deposition of a subaqueous delta in relation to the stillstand and slight fall of relative sea level after the middle Holocene. The boundary of the succession with the basal peat in core CM3 and Pleistocene basement in cores CM2 and CM4 is defined as the transgressive surface.

Radiocarbon dating

Accelerator mass spectrometry (AMS) radiocarbon dating of shells and plant fragments from the sediment cores was practiced by Beta Analytic Inc. (Table S2). The ages obtained were converted into calendar ages by the program CALIB 7.1.0 (Stuiver and Reimer, 1993) using the data set MARINE13.14c with a delta R of -60 ± 28 years for the shell samples and INTCAL13.14c for the plant fragment sample (Reimer et al., 2013).

The delta R was a weighted mean of data at the nearest two points (Southon et al., 2002; Dang et al., 2004). All ages are presented with 2 σ errors with the year 2015 at the datum.

Sample preparation, OSL measurements, and dose-rate determination

Sediments for OSL dating at the auger boreholes were obtained by hammering a light-tight stainless tube 20–30 cm long and 6.5 cm in diameter into sediment that had not been exposed to light. A shorter plastic tube 15 cm long and 5 cm in diameter was then hammered into the stainless tube for transport to the laboratories. Sample preparation and measurements for OSL dating were done at the luminescence laboratories of the University of Sheffield and Geological Survey of Japan. Samples were prepared under controlled red light to avoid affecting the quartz OSL signals. Sediment within 20–25 mm of the ends of the plastic tube was removed and used for measurements of water content and dosimetry. The remaining samples were processed for luminescence measurements. For sand samples obtained at beach-ridge sites, quartz grains representing a coarse fraction 120–180 μm in diameter were extracted from bulk samples following the method of Bateman and Catt (1996). Mud samples from the muddy plain were processed with hydrochloric acid, hydrogen peroxide, and settling cylinder to extract polymineral fine grains of 4–11 μm diameter. The fine quartz grains from these samples were further purified by processing the polymineral grains with hexafluorosilicic acid. Monolayers of quartz were mounted on 9.8 mm in diameter disks to form large (8 mm in diameter) aliquots, which were then measured with a TL-DA-20 automated Risø TL/OSL reader equipped with blue LEDs for stimulation and a $^{90}Sr/^{90}Y$ beta source for laboratory irradiation. Emitted OSL through a Hoya U-340 filter was measured with a photomultiplier.

The single-aliquot regenerative-dose (SAR) protocol was used to determine the equivalent dose (D_e) using the OSL response to a test dose to monitor and correct for sensitivity changes (Murray and Wintle, 2000). OSL measurements were made at 125 °C. Preheat plateau test and dose recovery test were carried out on samples Shfd12012 and gsj16191 by changing the preheat temperature from 160 to 260 300 °C in 20 °C increments (Fig. S1). Thus, preheat temperatures of 240 and 220 °C were chosen for the coarse and fine grain samples, respectively. A 160 °C cut-heat for the OSL response

to the test dose was used for all samples. To determine D_e, four regeneration points were measured including 0 Gy and a replicate of the first regeneration point, which was used to check whether the sensitivity correction procedure was performing adequately. Data from aliquots were rejected if recycling ratios were beyond 1.0 ± 0.1. Feldspar contamination was also checked by using the IR ratio test. 20 and 8 replicates per sample were measured for coarse and fine grain samples, respectively (Fig. S2; Table S3).

The contributions of both natural radioisotopes and cosmic radiation were considered for determination of the environmental dose rate (Table S3). Concentrations of potassium, uranium, thorium, and rubidium were quantified by inductively coupled plasma mass spectrometry and were converted to dose rate based on data from Adamiec and Aitken (1998) and Marsh et al. (2002). Past changes of moisture content are unknown, so an uncertainty margin of 5% was applied to the measured moisture content values. Cosmic dose rate was estimated based on Prescott and Hutton (1994). The attenuation factors used for beta and alpha rays were based on Mejdahl (1979) and Bell (1980), respectively. For fine grain samples, we used an a-value of 0.038 ± 0.002 (Rees-Jones, 1995). The final D_e value was determined by applying the Central Age Model (Galbraith et al., 1999) for individual samples. Statistical outliers were removed using a criterion that removes aliquots with D_e that falls outside the 25th and 75th percentiles (Fig. S2). D_e values were then divided by environmental dose rates to obtain OSL ages. All ages are expressed relative to AD 2016 (Table S3).

Reference

Adamiec, G. & Aitken M.J. Dose-rate conversion factors: update. *Ancient TL* **16,** 37–46 (1998).

Bateman, M. D. & Catt, J. A. An absolute chronology for the raised beach deposits at Sewerby, E. Yorkshire, UK. Jour. *Quat. Sci.* **11,** 389–395 (1996).

Bell W. T. Alpha dose attenuation in quartz grains for thermoluminescence dating. *Ancient TL* **12,** 4–8 (1980).

Dang, P. X., Mitsuguchi, T., Kitagawa, H., Shibata, Y. & Kobayashi, T. Marine reservoir correction in the south of Vietnam estimated from an annually-banded coral. *Radiocarbon* **46,** 657–660 (2004)

Galbraith, R. F., Roberts, R. G., Laslett, G. M., Yoshida, H. & Olley, J. M. Optical dating

of single and multiple grains of quartz from Jinmium rock shelter, northern Australia: Part I, experimental design and statistical models. *Archaeometry* **41,** 339–364 (1999).

Hanebuth, T. J. J., Voris, H. K., Yokoyama, Y., Saito, Y. & Okuno, J. Formation and fate of sedimentary depocentres on Southeast Asia's Sunda Shelf over the past sea-level cycle and biogeographic implications. *Earth-Sci. Rev.* **104,** 92–110 (2011).

Marsh, R. E., Prestwich, W. V., Rink, W. J. & Brennan, B. J. Monte Carlo determinations of the beta dose rate to tooth enamel. *Rad. Meas.* **35,** 609–616 (2002).

Mejdahl, V. Thermoluminescence dating: beta-dose attenuation in quartz grains. *Archaeometry* **21,** 61–72 (1979).

Murray, A. S. & Wintle, A. G. Luminescence dating of quartz using an improved single-aliquot regenerative-dose protocol. *Rad. Meas.* **32,** 57–73 (2000).

Prescott, J. R. & Hutton, J. T. Cosmic ray contribution to dose rates for luminescence and ESR dating: large depths and long-term time variations. *Rad. Meas.* **23,** 497–500 (1994).

Rees-Jones, J. Optical dating of young sediments using fine-grain quartz. *Ancient TL* **13,** 9–14 (1995).

Reimer, P. J. *et al.* IntCal13 and Marine13 radiocarbon age calibration curves 0–50,000 years cal BP. *Radiocarbon* **55,** 1869–1887 (2013).

Southon, J., Kashgarian, M., Fontugne, M., Metivier, B. & Yim, W. W-S. Marine reservoir corrections for the Indian Ocean and Southeast Asia. *Radiocarbon* **44,** 167–180 (2002).

Stuiver, M., Reimer, P. J. Extended ^{14}C database and revised CALIB radiocarbon calibration program. *Radiocarbon* **35,** 215–230 (1993).

Table S1. Locations of sediment cores obtained from the southwestern part of the Mekong River delta. amsl, Above the present mean sea level.

Core	Latitude (N, degree)	Longitude (E, degree)	Elevation (m, amsl)	Drilling depth (m)
ST3	9.4994	106.0303	+2	24
CM2	9.0120	105.1892	+1	26
CM3	8.8078	105.0061	+1	40
CM4	9.2350	105.4600	+1	18
CM5	9.0348	105.4291	+1.5	10
CM6	8.9691	105.3216	+1.5	22

Table S2 Radiocarbon dating results of plant fragments and Mollusc shells in the sediment cores ST3 and CM2–6. Core depth is relative to the ground at the individual core site. 2s calibrated age in ka (thousand years ago) relative to AD2015 is also shown, and those used in Fig. 2 are in Bold.

Access code	Core depth (m)	Sample	Conventional ^{14}C age (yr BP)	$\delta^{13}C$ (permil)	Calibrated age (1σ) (cal yr BP)	Probability	Calibrated age (2σ) (cal yr BP)	Probability	Calibrated age (2σ) (ka)
ST3									
Beta-326958	3.12	Mollusc shell	1880 ± 30	-2.9	1425-1539	1.000	1377-1596	1.000	**1.4-1.7**
Beta-326959	4.73	Mollusc shell	2160 ± 30	-1.3	1766-1878	1.000	1705-1925	1.000	**1.8-2.0**
Beta-326960	6.47	Mollusc shell	2470 ± 30	-1.0	2148-2272	1.000	2091-2311	1.000	**2.2-2.4**
Beta-326961	7.40	Mollusc shell	2500 ± 30	-2.6	2179-2296	1.000	2123-2327	1.000	**2.2-2.4**
Beta-326962	9.04	Mollusc shell	2330 ± 30	-1.3	1964-2086	1.000	1904-2133	1.000	**2.0-2.2**
Beta-326963	9.86	Mollusc shell	2500 ± 30	+1.1	2179-2296	1.000	2123-2327	1.000	**2.2-2.4**
Beta-326964	11.42	Mollusc shell	2830 ± 30	-3.0	2641-2732	0.921	2511-2748	1.000	**2.6-2.8**
					2614-2628	0.079			
Beta-326965	15.24	Mollusc shell	2800 ± 30	-1.4	2572-2709	1.000	2483-2729	1.000	**2.5-2.8**
Beta-326966	17.25	Mollusc shell	3150 ± 30	-2.7	2943-3079	1.000	2883-3148	1.000	**2.9-3.2**
Beta-326967	19.94	Mollusc shell	3390 ± 30	-2.5	3263-3377	1.000	3211-3432	1.000	**3.3-3.5**
Beta-326968	21.09	Mollusc shell	4160 ± 30	+1.3	4261-4390	1.000	4173-4427	1.000	**4.2-4.5**
Beta-326969	21.68	Mollusc shell	5220 ± 30	+0.3	5586-5673	1.000	5566-5732	1.000	5.6-5.8
Beta-326977	21.85	Mollusc shell	4440 ± 30	+1.0	4642-4784	1.000	4564-4813	1.000	**4.6-4.9**
Beta-326157	22.20	Plant fragment	6970 ± 40	-27.4	7738-7849	1.000	7695-7870	0.907	7.8-7.9
							7897-7926	0.093	
Beta-328105	22.58	Mollusc shell	7060 ± 40	-2.5	7554-7643	1.000	7494-7684	1.000	**7.6-7.7**
Beta-326978	22.80	Mollusc shell	10940 ± 50	-4.2	12501-12617	1.000	12399-12684	1.000	12.5-12.7
Beta-326158	23.65	Plant fragment	6690 ± 40	-29.4	7557-7592	0.557	7478-7622	1.000	**7.5-7.7**
					7511-7542	0.443			
Beta-326159	23.94	Plant fragment	6840 ± 50	-25.9	7614-7707	1.000	7588-7764	0.973	7.7-7.8
							7770-7786	0.027	
CM-2									
Beta-399868	0.77	Plant fragment	210 ± 30	-26.3	150-174	0.410	144-216	0.535	**0.21-0.28**
					273-298	0.370	305	0.324	
					0-10	0.147	0-21	0.141	
					178-185	0.073			
Beta-399869	2.79	Plant fragment	2360 ± 30	-29.7	2341-2380	0.747	2332-2468	0.992	2.4-2.5
					2393-2423	0.253	2478-2483	0.008	
Beta-399870	4.63	Plant fragment	450 ± 30	-27.7	498-521	1.000	472-535	1.000	**0.54-0.60**
Beta-401925	9.36	Mollusc shell	1590 ± 30	+1.0	1175-1258	1.000	1113-1292	1.000	**1.2-1.4**
Beta-399872	11.74	Mollusc shell	1880 ± 30	-1.6	1425-1539	1.000	1377-1596	1.000	**1.4-1.7**
Beta-401926	12.95	Mollusc shell	1970 ± 30	+0.5	1539-1651	1.000	1497-1711	1.000	**1.6-1.8**
Beta-401927	16.20	Mollusc shell	3350 ± 30	-0.7	3226-3338	1.000	3160-3380	1.000	**3.2-3.4**
Beta-401928	17.32	Mollusc shell	3360 ± 30	-0.9	3236-3347	1.000	3170-3392	1.000	**3.2-3.5**
Beta-401929	19.23	Mollusc shell	3420 ± 30	-0.3	3318-3422	1.000	3238-3461	1.000	**3.3-3.5**
Beta-401930	20.77	Mollusc shell	3370 ± 30	-0.5	3245-3356	1.000	3180-3405	1.000	**3.2-3.5**
CM-3									
Beta-399874	2.60	Plant fragment	130 ±30	-29.3	66-118	0.384	56-151	0.444	**0.12-0.22**
					243-268	0.177	173-275	0.399	
					14-37	0.166	9-45	0.157	
					125-146	0.130			
					213-231	0.127			
					189-192	0.017			
Beta-399875	5.45	Plant fragment	120 ±30	-28.7	62-119	0.452	11-149	0.659	**0.08-0.21**
					241-265	0.173	186-271	0.341	
					123-142	0.141			
					23-40	0.128			
					219-232	0.106			
Beta-401931	8.99	Mollusc shell	1070 ± 30	-2.2	648-715	1.000	620-769	1.000	**0.69-0.83**
Beta-399876	11.88	Mollusc shell	1100 ± 30	-4.6	663-737	1.000	637-790	1.000	**0.70-0.86**
Beta-401932	17.85	Mollusc shell	1230 ± 30	0.0	787-889	1.000	733-920	1.000	**0.80-0.99**
Beta-401933	19.72	Mollusc shell	1310 ± 30	+0.9	872-967	1.000	797-1015	1.000	**0.86-1.1**
Beta-401940	20.12	Mollusc shell	3410 ± 30	+0.3	3302-3413	1.000	3229-3449	1.000	3.3-3.5
Beta-401934	21.90	Mollusc shell	1300 ± 30	-0.9	857-956	1.000	787-999	1.000	**0.85-1.1**
Beta-401935	27.79	Mollusc shell	3660 ± 30	-2.1	3575-3693	1.000	3534-3795	1.000	**3.6-3.9**
Beta-401936	29.63	Mollusc shell	8070 ± 30	-4.9	8531-8653	1.000	8474-8746	1.000	8.5-8.8
Beta-401937	30.80	Mollusc shell	8120 ± 30	-4.4	8587-8731	1.000	8542-8846	1.000	8.6-8.9
Beta-401938	32.20	Mollusc shell	3060 ± 30	+1.3	2834-2955	1.000	2774-3019	1.000	**2.8-3.1**
Beta-399878	32.58	Plant fragment	8840 ± 30	-27.8	10064-10121	0.328	9743-9961	0.591	9.8-10.0
					9883-9936	0.299	10055-10152	0.301	
					9785-9849	0.265	9986-10043	0.108	
					9860-9878	0.070			
					9995-10005	0.038			

Beta-401939	32.60	Mollusc shell	8250 ± 30	-4.2	8800-8826	1.000	8712-8994	1.000	8.8-9.1
Beta-399879	32.69	Plant fragment	9040 ± 40	-29.3	10198-10233	1.000	10173-10249	1.000	**10.2-10.3**
Beta-399880	33.01	Plant fragment	9030 ± 40	-28.0	10194-10231	1.000	10163-10248	1.000	10.2-10.3
Beta-399881	33.35	Plant fragment	8890 ± 40	-28.1	9981-10080	0.534	9887-10187	0.984	10.0-10.3
					9923-9971	0.249	9819-9840	0.012	
					10115-10155	0.217	9797-9803	0.003	
							9869-9871	0.001	
Beta-399882	33.71	Plant fragment	8930 ± 40	-29.4	10128-10185	0.374	9914-10099	0.654	**10.0-10.2**
					9940-9992	0.336	10109-10198	0.346	
					10035-10062	0.168			
					10008-10030	0.122			
CM-4									
Beta-442061	1.41	Mollusc shell	1670 ± 30	-1.6	1246-1322	1.000	1198-1367	0.996	1.3-1.4
							1189-1192	0.004	
Beta-440363	2.53	Plant fragment	820 ± 30	-26.5	691-744	0.988	686-784	1.000	**0.75-0.85**
					754-756	0.012			
Beta-442062	2.72	Mollusc shell	1730 ± 30	-2.0	1287-1369	1.000	1254-1435	1.000	1.3-1.5
Beta-442063	5.34	Mollusc shell	1810 ± 30	-3.6	1360-1475	1.000	1316-1518	1.000	**1.4-1.6**
Beta-442064	9.16	Mollusc shell	3840 ± 30	-0.1	3820-3940	1.000	3723-3998	1.000	**3.8-4.1**
Beta-442065	14.79	Mollusc shell	3960 ± 30	-0.6	3963-4095	1.000	3894-4160	1.000	**4.0-4.2**
Beta-442066	16.35	Mollusc shell	4260 ± 30	+0.6	4401-4507	1.000	4322-4574	1.000	**4.4-4.6**
Beta-442067	17.42	Mollusc shell	4490 ± 30	-7.0	4706-4821	1.000	4616-4846	1.000	4.7-4.9
CM-5									
Beta-440364	1.87	Plant fragment	460 ± 30	-27.0	502-523	1.000	484-537	1.000	**0.55-0.60**
Beta-440365	3.90	Plant fragment	1240 ± 30	-26.8	1091-1108	0.109	1169-1266	0.644	**1.2-1.3**
					1146-1158	0.259	1073-1163	0.356	
					1200-1261	0.632			
Beta-442068	7.83	Mollusc shell	1440 ± 30	-4.5	986-1108	1.000	949-1163	1.000	**1.0-1.2**
Beta-442069	9.60	Mollusc shell	1540 ± 30	-2.0	1117-1227	1.000	1057-1257	1.000	**1.1-1.3**
CM-6									
Beta-440366	2.82	Plant fragment	330 ± 40	-26.0	315-335	0.171	305-484	1.000	**0.37-0.55**
					349-410	0.550			
					420-455	0.279			
Beta-442070	8.49	Mollusc shell	1480 ± 30	-2.8	1048-1160	1.000	980-1208	1.000	**1.0-1.3**
Beta-442071	11.62	Mollusc shell	1710 ± 30	-0.5	1275-1351	1.000	1240-1404	1.000	**1.3-1.5**
Beta-442072	13.72	Mollusc shell	1760 ± 30	-1.0	1303-1399	1.000	1279-1477	1.000	**1.3-1.5**
Beta-442073	14.25	Mollusc shell	2190 ± 30	-0.5	1805-1912	1.000	1736-1965	1.000	1.8-2.0
Beta-442074	18.20	Mollusc shell	1540 ± 30	+0.2	1117-1227	1.000	1057-1257	1.000	**1.1-1.3**
Beta-442075	21.94	Mollusc shell	5330 ± 30	-3.0	5708-5837	1.000	5650-5876	1.000	**5.7-5.9**
MRD-505									
Beta-442060	1.80	Mollusc shell	2810 ± 30	-0.7	2579-2581	0.005	2492-2734	1.000	2.6-2.8
					2589-2591	0.005			
					2595-2720	0.990			

Table S3. OSL samples and dating results: coordinate of sampling point, depth of sample from the ground, size of extracted grain, contents of radionuclides, water content, cosmic dose rate, environmental dose rate, equivalent dose (*De*), overdispersion value (OD) after the Central Age Model, numbers of aliquots measured and used for the determination of *De*, and OSL age. OSL ages with standard error exceeding 10 years are rounded to the nearest decade.

Site	Lab code	Latitude (N, degree)	Longitude (E, degree)	Depth (cm)	Grain size (μm)	K (%)	U (ppm)	Th (ppm)	Rb (ppm)	Water content (%)	Cosmic dose rate (mGy/ka)	Total dose rate (mGy/ka)	D_e (mGy)	OD (%)	No. aliquots measured	No. aliquots used	Age (yr BP)
MRD-301	Shfd12001	9.66842	105.95161	110–125	120–180	1.07	1.46	9.02	66.1	16	167±8	1919±90	5321±29	5	20	16	**2770±130**
MRD-304	Shfd12002	9.62928	105.95169	87–102	120–180	1.45	1.09	6.58	76.1	17	172±9	2005±103	4977±42	5	20	17	**2480±130**
MRD-307	Shfd12003	9.56073	105.94508	108–123	120–180	1.04	2.26	15.30	66.7	16	167±8	2446±112	4614±36	6	20	16	**1890±90**
MRD-310	Shfd12004	9.52549	105.96008	115–130	120–180	1.28	1.29	8.86	70.2	14	165±8	2084±102	2849±25	10	20	17	**1370±70**
MRD-311	Shfd12005	9.40677	106.05959	103–118	120–180	1.29	1.58	9.71	80.1	16	168±8	2171±104	1306±10	6	20	13	**600±30**
MRD-316	Shfd12006	9.37276	106.11057	110–125	120–180	1.23	1.22	6.85	75.8	17	167±8	1864±92	1105±9	6	20	14	**590±30**
MRD-320	Shfd12007	9.35896	106.06247	91–106	120–180	1.17	2.55	17.90	77.2	15	171±9	2822±131	1303±8	3	20	15	**460±20**
MRD-322	Shfd12008	9.35221	106.05056	97–112	120–180	1.25	1.15	6.40	80.7	16	170±8	1854±92	883±10	6	20	19	**480±20**
MRD-325	Shfd12009	9.38308	106.15474	100–115	120–180	0.91	6.93	57.50	58.9	15	169±8	5951±302	944±17	9	20	19	**160±10**
MRD-328	Shfd12010	9.34641	106.10588	109–124	120–180	1.09	1.25	7.24	70.7	17	167±8	1778±85	358±8	6	20	17	**200±10**
MRD-330	Shfd12011	9.32639	106.00008	112–127	120–180	1.09	1.15	6.58	67.6	16	166±8	1723±83	630±9	5	20	19	**370±20**
MRD-332	Shfd12012	9.30978	105.98524	108–123	120–180	0.83	3.00	20.10	54.6	16	167±8	2734±127	338±8	0	20	15	**120±10**
MRD-333	Shfd12013	9.33589	106.07742	113–128	120–180	1.00	1.87	12.40	64.0	16	166±8	2162±99	306±7	0	20	20	**140±10**
MRD-501	gsj16186	9.48516	105.28192	175-190	4–11	1.6	3.75	15	128	48	153±15	3490±193	7935±75	0	8	8	**2690±150**
MRD-502	gsj16187	9.52373	105.24624	175-190	4–11	1.7	3.67	14.9	117	46	153±15	3637±203	6948±64	0	8	8	**2230±130**
MRD-503	gsj16189	9.58506	105.19962	160-175	4–11	1.5	3.68	13.9	97.5	42	156±16	3467±198	5737±64	2	8	7	**1970±110**
MRD-504	gsj16190	9.14195	105.12775	175-190	4–11	2.2	2.84	15.5	137	42	153±15	3901±222	2884±27	0	8	6	**860±50**
MRD-505	gsj16191	9.27422	105.20190	190-205	4–11	1.7	4.42	13.9	116	39	151±15	3941±230	5101±52	1	8	8	**1540±90**
MRD-506	gsj16192	9.32785	105.20388	185-200	4–11	1.7	2.99	15.4	118	42	152±15	3574±203	5996±59	1	8	7	**1970±110**
CM-4	gsj16193	9.23498	105.46003	260-265	4–11	2.2	2.81	12.7	136	41	140±14	3588±204	3347±31	0	8	6	**1070±60**

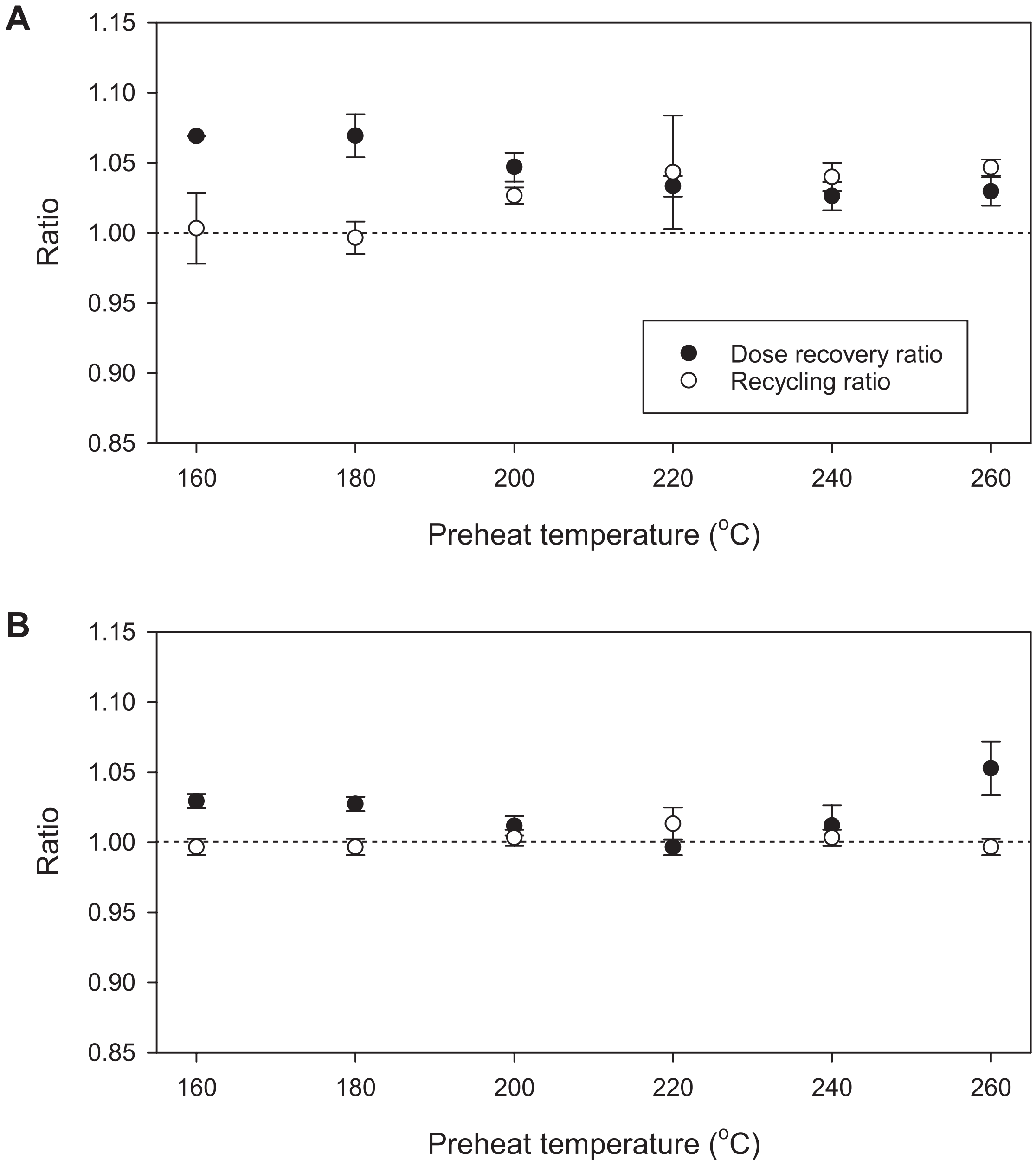

Fig. S1. Results of dose recovery test on (A) Shfd12012 and (B) gsj16191 with different preheat temperatures. Recycling ratios observed in the test are also shown.

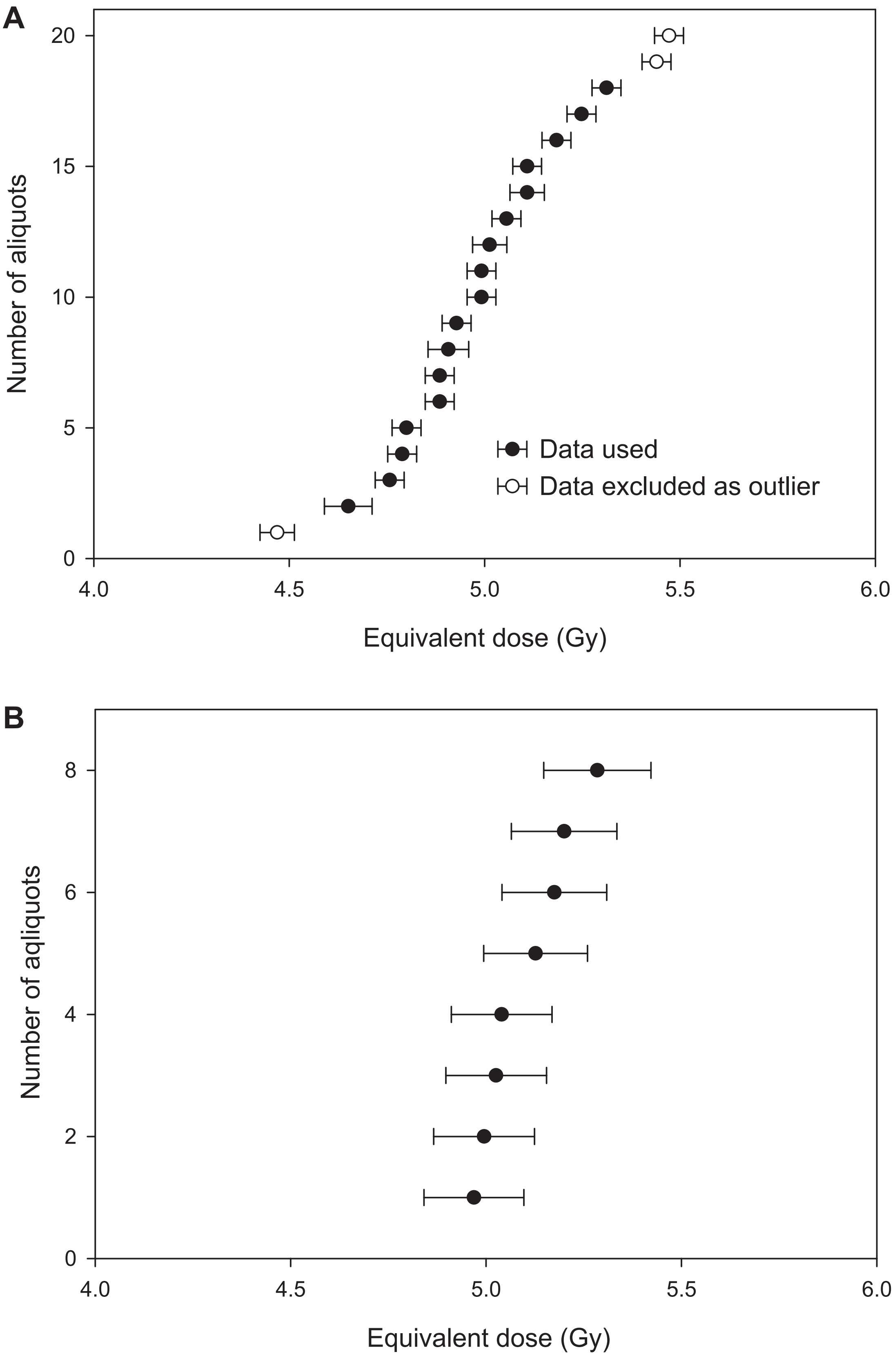

Fig. S2. Distributions of equivalent dose determined for samples (A) Shfd12002 and (B) gsj16191.
